# Divergent functions of the RNA-binding protein, Mei-P26 in germline and somatic lineages of *Drosophila* testis

**DOI:** 10.3389/fcell.2026.1739650

**Published:** 2026-02-18

**Authors:** Shallinie Thangadurai, Elena S. Pak, Alexander K. Murashov

**Affiliations:** Department of Comparative Biomedical Sciences, School of Veterinary Medicine, Louisiana State University, Baton Rouge, LA, United States

**Keywords:** cell cycle, Mei-P26, RNA-binding protein, spermatogenesis, TOR signaling

## Abstract

The *Drosophila* TRIM-NHL family RNA-binding protein Mei-P26 has been implicated in suppressing tumorigenesis in the female germline. By restricting growth and proliferation while promoting differentiation within the ovarian stem cell lineage, Mei-P26 maintains germline homeostasis. Whether it plays comparable or distinct roles in the testis during spermatogenesis, however, remains unclear. Here we show that overexpression of *mei-P26* in germline cells leads to truncated, agametic testes. These testes display germline cell cycle arrest and defective somatic cyst cell proliferation and differentiation, highlighting a failure in germline-soma coordination. By contrast, somatic overexpression of *mei-P26* results in male-specific lethality. With the *Gal80* ^*ts*^ system to circumvent this lethality, induction of *mei-P26* in adult somatic cells promoted proliferation and differentiation of both germline and somatic cells, accompanied by upregulation of phospho-S6, indicating hyperactivation of the TOR signaling pathway. Our findings reveal that Mei-P26 exerts opposing, cell type-specific roles within the same tissue, restraining germline development while driving expansion of somatic cells. This study underscores the importance of cell-specific regulation by RNA-binding proteins and provides new insights into how misregulation of Mei-P26 may coordinate divergent cell fates within a shared tissue context-processes frequently disrupted in cancer, developmental disorders, and age-associated tissue decline.

## Introduction

Stem cells are responsible for the constant supply of highly differentiated, short-lived cells, such as sperm, endocrine cells, and eggs (Kiger et al., 2000). Adult stem cells in regenerative tissues undergo mitotic divisions to replenish lost, damaged, or terminally differentiated cells. To maintain tissue homeostasis through sufficient production of differentiated cells and reduce the risk of oncogenic mutations from excessive proliferation, stem cell progeny usually undergoes several rounds of transit-amplifying (TA) divisions before terminal differentiation. However, failure to properly regulate the termination of transit-amplifying (TA) cell divisions and to initiate final differentiation can result in abnormal cell proliferation, which may contribute to the development of tumors in adult stem cell lineages (Chen et al., 2014).

The niche within the *Drosophila melanogaster* testis constitutes a specialized microenvironment that includes germline stem cells (GSCs) and somatic cyst stem cells (CySCs), both of which are anchored to a cluster of post-mitotic somatic cells called the ‘hub’. This setup serves as a powerful model for studying stem cell biology (Singh et al., 2016). GSCs undergo asymmetric division where one daughter cell gains stem cell status and remain in the niche, while the other daughter cell differentiates into gonialblast (GB) and migrate away from the niche. This GB undergoes four rounds of TA divisions with incomplete cytokinesis, resulting in a cluster of 16 interconnected spermatogonial cells that develop synchronously. This process is then followed by the initiation of the meiotic program to produce spermatocytes. The second stem cell population, somatic cyst stem cells (CySCs), generates post-mitotic differentiating progeny known as cyst cells. These cyst cells establish a stable 2:1 association with each gonialblast, encapsulating the developing germline cyst and ultimately forming a physical barrier that isolates it from the surrounding microenvironment. Proper coordination between germline and somatic lineages, along with somatic support, is crucial for the progression of germ cell differentiation and the maintenance of fertility (Nagai et al., 2022; Yuen et al., 2021).

During spermatogenesis, many signaling pathways are regulated strictly to determine the fate of the stem cells. Hub cells secrete cytokine called Unpaired (Upd), which then activates the JAK-STAT signaling in the adjacent GSCs and CySCs. Bone morphogenetic protein (BMP) regulates the GSC self-renewal, while chronologically inappropriate morphogenesis (chinmo), a target of activated STAT, is required for the self-renewal of CySCs. Expression of bag of marbles (Bam) promotes the differentiation of the GSCs into gonialblasts and spermatogonia (de Cuevas and Matunis, 2011; Hasan et al., 2015; Matunis et al., 2012). Coordinated regulation of the somatic lineage is equally critical, as differentiation of cyst cells requires PI3K/Tor pathway activity (Amoyel et al., 2016a; Wang C. et al., 2025). Together, these pathways ensure the synchronized progression of germline and somatic cells, which is essential for proper spermatogenesis.

Mei-P26, a TRIM-NHL (tripartite motif and Ncl-1, HT2a, and Lin-41 domain) family meiotic protein, regulates Bam protein accumulation and subsequently acts as a direct target of translational repression by Bam and Bgcn in germ cells. Mei-P26 plays a pivotal role in promoting the mitosis-to-meiosis transition and facilitating the subsequent differentiation of spermatocytes (Insco et al., 2009; 2012). Loss-of-function mutations in Mei-P26 lead to tumorigenic over-proliferation in both the ovary and testis, marked by the accumulation of undifferentiated, multicellular germline cysts (Page et al., 2000). Mei-P26 is a tumour suppressor protein, and this action is exerted in part via the suppression of dMyc, an oncogene that promotes cell growth and ribosome biogenesis (Ferreira et al., 2014). Additionally, Mei-P26 activates TOR signaling pathway in the female GSCs to induce cell differentiation (Gui et al., 2023). These phenotypes underscore the critical role of *mei-P26* in regulating both stem cell maintenance and stem cell daughter differentiation (Li et al., 2012).

Emerging evidence suggests that GSCs division and differentiation are not solely governed by cell-intrinsic mechanisms but are also influenced by extrinsic cues derived from adjacent CySCs (Smendziuk et al., 2015). While the role of *mei-P26* in the *Drosophila* germline has been extensively characterized, recent findings demonstrating its expression in non-germline tissues (Hu et al., 2025) prompted us to investigate whether *mei-P26* expression in germline-adjacent somatic cells of the testis contributes to developmental signaling pathways that regulate both germline and somatic cell fates.

To address this, we examined *mei-P26* function in both germline and somatic cells of the *Drosophila* testis. Germline overexpression of *mei-P26* resulted in truncated, agametic testes with germline cell-cycle arrest and defective cyst cell differentiation, indicating disrupted germline-soma coordination. In contrast, somatic overexpression enhanced proliferation and differentiation through TOR pathway activation. These findings demonstrate that Mei-P26 exerts opposing roles in distinct cellular compartments, restricting germline proliferation while promoting somatic growth, revealing its critical function in maintaining communication and balance between the germline and soma.

## Materials and methods

### Fly strains

All flies were maintained on the standard Bloomington Formulation diet (Nutri-Fly® BF, Cat #: 66–112, Genesee Scientific Inc.) in a climate-controlled environment at 24 °C under a 12 h light-dark cycle and 65%–70% humidity. Crosses with *GAL80* ^*TS*^, which were maintained at 18 °C until eclosion, and the adult flies were transferred to 29 °C. Flies were reared at the permissive temperature (18 °C) and adult males were collected and shifted to the restrictive temperature of 29 °C to allow GAL4 activity. Rescue experiments without GAL80 were raised at room temperature until eclosion and shifted to 26 °C for the specified period to ensure maximum GAL4 activity. *nos-GAL4::VP16* dependent *mei-P26* overexpression phenotype is not suppressible with GAL80. Therefore, to ensure that the phenotype observed with germline-specific overexpression and somatic cell-specific overexpression is only optimally induced in adulthood, crosses with nos-GAL4 were also made at 18 °C until eclosion and then adult male F1s were transferred to 26 °C prior to dissection.

Fly strains used in this research were obtained from Bloomington *Drosophila* Stock Center (BDSC) and RNAi lines were from the Vienna *Drosophila* RNAi Center (VDRC). The following fly lines were used in this study: *w* ^1118^ (BDSC# 3605), *UAS-mei-P26* (BDSC# 25771, #25772), *c587-GAL4* (BDSC# 67747), *nos-GAL4::VP16* (BDSC# 64277), *UAS-mei-P26-RNAi* (VDRC# 101060).

### Immunostaining

The testes of aged (5–7 days after eclosion) unmated F1s with desired genotype were dissected in ice cold PBSTX (1X PBS with 0.2% Triton X-100, 0.1% Tween-20). The dissected testes were fixed in 4% paraformaldehyde (PFA) for 30 min, washed thrice with PBSTX, and blocked in PBSTX +5% NGS (5% normal goat serum) in PBSTX for an hour. The blocked samples were then nutated with primary antibodies at 4 °C overnight. The testes samples were then washed thrice in PBSTX, followed by blocking for 1 hour in PBSTX +5% NGS before incubating with secondary antibodies at room temperature for 2.5 h. The primary antibodies used in this study were guinea pig anti-Traffic jam (1:400; a kind gift from Dorothea Godt, University of Toronto) (Li et al., 2003), rabbit anti-Mei-P26 (1:400; a kind gift from Paul Lasko, McGill University) (Liu et al., 2009), mouse anti-1B1 (HTS; 1:50; Developmental Studies Hybridoma Bank [DSHB]), mouse anti-α-Spectrin (α-Spec; 1:100; DSHB) mouse anti-dMyc (1:1; DSHB), mouse anti-Eya (1:10; DSHB), mouse anti-LamC (1:50; DSHB), rat anti-Vasa (1:10; Santa Cruz), rabbit anti-Phospho-Histone H3 (Ser10; 1:500, Invitrogen), rabbit anti-Cleaved Caspase-3 (1:400; Cell Signaling Technology) rabbit anti-pSmad (1:100; Cell Signaling Technology) and rabbit anti-Phospho-S6 Ribosomal Protein (Ser235/236; 1:800, Cell Signaling Technology). The secondary antibodies used in this study were goat anti-rabbit IgG Alexa Fluor 488, goat anti-mouse IgG Alexa Fluor 488, goat anti-mouse IgG Alexa Fluor 594, goat anti-guinea pig IgG Alexa Fluor 488, and goat anti-guinea pig IgG Alexa Fluor 594 (Invitrogen). All secondary antibodies were used at dilution of 1:500. Then, the testes samples were washed thrice in PBSTX and mounted on glass slide with 10 μL SlowFade mounting medium with DAPI (Biotium).

### BrdU proliferation assay

Bromodeoxyuridine (BrdU) is a S-phase marker which is used to identify proliferating cells. The adult aged unmated male F1 flies with desired genotype were starved for 2 h before feeding with 2.5 mM BrdU (SIGMA, Germany) in DMSO with 6% (v/v) red food coloring to monitor feeding for 24 h at 26 °C (Singh et al., 2016). Flies that have red abdominal pigmentation were selected and the testes were dissected immediately for BrdU labelling. The protocol described by Brawley and Matunis was followed with a slight modification (Brawley and Matunis, 2004). Briefly, the testes were fixed using 4% PFA immediately after dissection for 30 min and DNA was subsequently denatured using 2N HCl for 30 min (Boyle et al., 2007). This is followed by standard immunostaining protocol mentioned above using mouse anti-BrdU (1:100; DSHB) primary antibody, and goat anti-mouse AlexaFluor 594 (1:500; Invitrogen) secondary antibodies. The number of BrdU labelled cells were counted.

### TUNEL apoptosis detection assay

Apoptotic cells were detected using the terminal deoxynucleotidyl transferase dUTP nick end labeling (TUNEL) assay with the OneStep TUNEL Apoptosis Kit [Red, 594] (Novus Biologicals; Cat. No. NBP3-11959). Male unmated F1s with desired genotype were dissected in PBSTX followed by immediate fixing for 30 min in 4% PFA, washed thrice, and labelled according to the manufacturer’s instructions with slight modification. Briefly, after washing, fixed testes samples were incubated in the TdT buffer for 30 min at 37 °C. Then the samples were nutated in dark at 37 °C for at least 2 h with a cocktail of TdT buffer, staining solution and terminal deoxynucleotidyl transferase (TdT). The samples were washed thrice (15 min each) in dark. Then the samples were directly mounted on glass slide with SlowFade mounting medium with DAPI (Biotium) as described above or standard immunostaining protocol was followed for counterstaining.

### RNA isolation and quantitative real-time PCR

Adult unmated males of 5–7 days in age were dissected quickly in filter sterilized ice cold PBSTX. A maximum of thirty males were processed per round. As much of the dissecting medium was removed prior to the addition of 100 μL of TRIzol (Invitrogen). Tubes were immediately placed in the −80 °C refrigerator for flash freezing. Three batches of testes (60 pairs each batch) were consolidated for a singular RNA extract. Total RNA from tissue samples was extracted with an RNAqueous Micro Kit (Thermo Fisher–Life Technologies) according to the manufacturer’s instructions. First-strand cDNA was synthesized from total RNA using the SuperScript™ VILO™ cDNA Synthesis Kit (Thermo Fisher Scientific; Cat. No. 11754250). Real-time PCR reaction on cDNA was performed using the TB Green Advantage qPCR Premix Kit (Takara Bio; Cat. No. 639676) on qTOWER^3^ iris 384 PCR System (Analytik Jena) according to the manufacturer’s instructions.

The following primers were used: *mei-P26*-specific Fw-5′-TCCGGGGATTCCCAATCTGAA and Rev-5′-GGAGCTAGAGCTGCTAGAACT, *RpL32*-specific Fw-5′-GACGCTTCAAGGGACAGTATCTG and Rev-5′- AAA​CGC​GGT​TCT​GCA​TGA​G which was used as a reference gene.

### Imaging, processing and quantification

Images were acquired using an Olympus FV3000 laser-scanning confocal microscope controlled by CellSens software (Olympus). Z-stack images were obtained using an Olympus fluorescence microscope (Olympus). Acquired images were converted to grayscale, and the number of cells and fluorescence intensity were quantified using ImageJ software. All image adjustments, i.e., brightness and contrast were applied uniformly across experimental groups.

### Statistical analysis

Samples were collected from at least five independent males grown under equivalent environmental conditions. Statistical significance between control and experimental groups was determined using the non-parametric Mann–Whitney U test. For comparisons between more than two datasets, a non-parametric Kruskal–Wallis one-way analysis of variance test followed by a Dunn’s multiple correction was used. Significance levels are indicated as follows: * = *p* < 0.05; ** = *p* < 0.005; *** = *p* < 0.0005; **** = *p* < 0.0001. All statistical analyses were performed using GraphPad Prism 10 software.

## Results

### Germline Mei-P26 overexpression disrupts testis development

**FIGURE 1:**
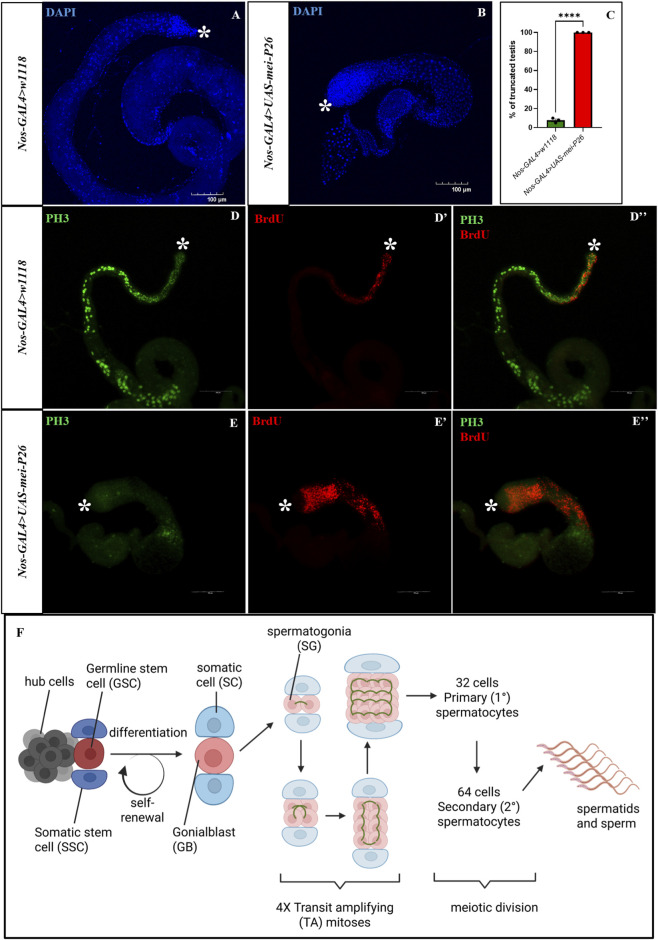
Germline *mei-P26* overexpression causes truncated testis formation. **(A)** Normal control testis, and **(B)** germline *mei-P26* overexpressed truncated testis with DAPI in blue. **(C)** Penetrance in percentage of truncated testis recovered. S-phase marked by BrdU in red and mitosis marked by PH3 in green to show proliferation and differentiation in the **(D–D”)** control testis and **(E–E”)** germline *mei-P26* overexpressed truncated testis. **(F)** Schematic diagram showing spermatogenesis in *Drosophila melanogaster*. The testis houses germline stem cells (GSCs) and somatic stem cells (SSCs) anchored to the quiescent hub cells. Each stem cell will undergo asymmetric division of self-renewal and differentiation. GSC will form gonialblast (GB), which then undergoes 4 rounds of transit amplifying (TA) mitotic division called endoreplication with incomplete cytokinesis, with cells held together by fusomes. These spermatogonia (SGs) then differentiate into spermatocytes after meiotic division and then proceed to further differentiate into spermatids and sperm. Throughout all these processes somatic cells (SCs) will encapsulate them by keeping them protected until terminal differentiation. Image frame: 100 μm. * indicates apical tip of the testis.

To explore how increased expression of *mei-P26* impacts male germline cells in *Drosophila*, we overexpressed *mei-P26* in the germline using nos-GAL4. We found that nos > *mei-P26* expressing flies exhibited shorter, truncated testes (n = 65) compared to nos > w1118 control males (n = 70), with 100% penetrance, as observed across three independent biological replicates (Figures 1A–C). Although nos > w1118 control flies exhibit a low baseline incidence of truncated testes (∼10%), this phenotype is substantially more penetrant and severe upon *mei-P26* overexpression. A similar developmental defect was observed in females, where *mei-P26* overexpression caused rudimentary ovarian formation in the unmated females (Supplementary Figure S1). These findings suggested an arrest or reduction in germ cell proliferation during gonadal development. To determine if testis truncation can be rescued by knocking down *mei-P26* in the germline alongside overexpression, we used a genetic fly construct containing *mei-P26* RNAi combined with UAS-*mei-P26*. Knocking down *mei-P26* alone was sufficient to suppress approximately 50% (n=60) of the testis truncation caused by *mei-P26* overexpression in the germline (Supplementary Figures S2A, B).

To assess cell proliferation and differentiation status in these testes, S-phase cells were labeled with BrdU (bromodeoxyuridine), and mitotic cells were identified using phospho–Ser10 histone H3 (PH3). Germline-specific overexpression of *mei-P26* showed an increase in BrdU-positive cells (Figure 1E’) compared to control testes (Figure 1D’), but the PH3-positive mitotic cells were markedly reduced and almost absent (Figure 1E) compared to control testes with PH3-positive mitotic cells (Figure 1D), indicating defective spermatogenesis where cells are arrested in the S-phase of the cell cycle. The *Drosophila* testis is tightly regulated by both cell-autonomous and non-cell-autonomous signaling to maintain controlled division and differentiation, especially at the transit-amplifying (TA) region where mitotic endoreplication occurs to produce enough spermatogonia that will further differentiate into spermatocytes through meiotic division. The endoreplication stage is a critical point where cells can accumulate damage during the four rounds of division and determine whether to enter meiosis, as only high-quality germ cells are committed to becoming sperm (Figure 1F) (Fabian and Brill, 2012).

### Germline overexpression of Mei-P26 causes meiotic arrest and suppresses BMP signaling

Previous studies have demonstrated that Mei-P26 plays a pivotal role in balancing cell proliferation and differentiation within the *Drosophila* germline. It acts downstream of the bam pathway to limit the proliferative potential of germline stem cells (GSCs) and their progeny, while promoting the onset of differentiation through post-transcriptional regulation of target mRNAs (Insco et al., 2012; Neumüller et al., 2008). Consistent with its role as a tumor suppressor in the germline, loss of *mei-P26* results in excessive proliferation of undifferentiated germ cells, whereas its overexpression can disrupt germline homeostasis and differentiation programs (Li et al., 2012; Page et al., 2000).

**FIGURE 2:**
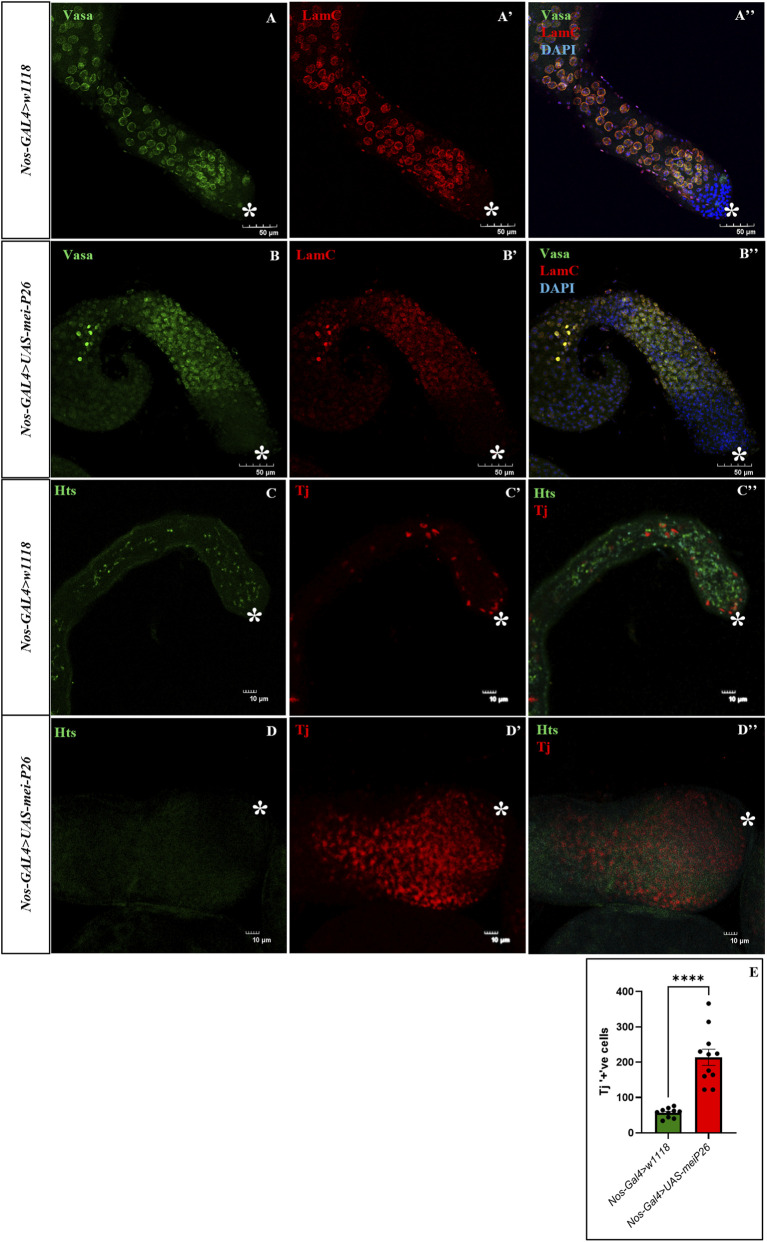
Germline coupled with somatic cell proliferation with no germline interconnecting fusomes in germline *mei-P26* overexpressed truncated testis. **(A,B)** Germline cells are marked by Vasa (in green), and spermatocytes are marked by LamC (in red). **(B–B”)** *Nos>UAS-mei-P26* has proliferation of germ cells and differentiated spermatocytes compared to control **(A–A”)**. However, these testes **(D–D”)** lack fusomes that interconnect germline cysts marked by Hu-li tai shao (Hts) (in green) **(D)**, although there is evident proliferation of Traffic jam (Tj) marked (in red) somatic cells **(D’)**, indicating premature differentiation of the germ cells due to loss of fusomes compared to control **(C–C”)** with interconnected branched fusomes. **(E)** Quantification of Tj+ cells indicates increased proliferation of somatic cells in the mutant testis. Image frame: **(A–B”)** 50μm, (C, D”) 10μm. * indicates the apical tip of the testis.

In our study, we observed that proliferating cells in the testes include both germline- and somatic-derived populations. This was evidenced by strong expression of the germline marker Vasa (Figure 2B) and the somatic cell marker Traffic Jam (Tj; Figure 2D’), in contrast to control testes, which showed Vasa expression restricted to germline cells (Figure 2A) and Tj expression restricted to somatic cells (Figure 2C’). The number of somatic cells undergoing TA divisions was quantified by counting Tj-positive cells and was significantly increased in germline-specific *mei-P26* overexpressing testes compared to controls (Figure 2E), consistent with the elevated proliferation detected by BrdU S-phase labeling (Figure 1E’). No fusome structures were observed in these testes (Figure 2D) when stained with an adducin homolog, Hu-li tai shao (Hts), indicating there are no clustered germline cysts as seen in the control testis (Figure 2C). Nonetheless, the detection of Vasa-positive germline cells without fusomes suggests a failure in incomplete cytokinesis during TA division that forms spermatogonia cysts. Interestingly, the somatic cells showed excessive proliferation, resembling a tumor-like phenotype within the testis. The absence of differentiating spermatogonia may deprive somatic cells of essential germline-derived differentiation cues, leading to their arrest at the TA division stage, in agreement with prior studies demonstrating that germline-to-soma signaling is necessary for proper somatic differentiation during testis development (Kiger et al., 2001; Sarkar et al., 2007).

**FIGURE 3:**
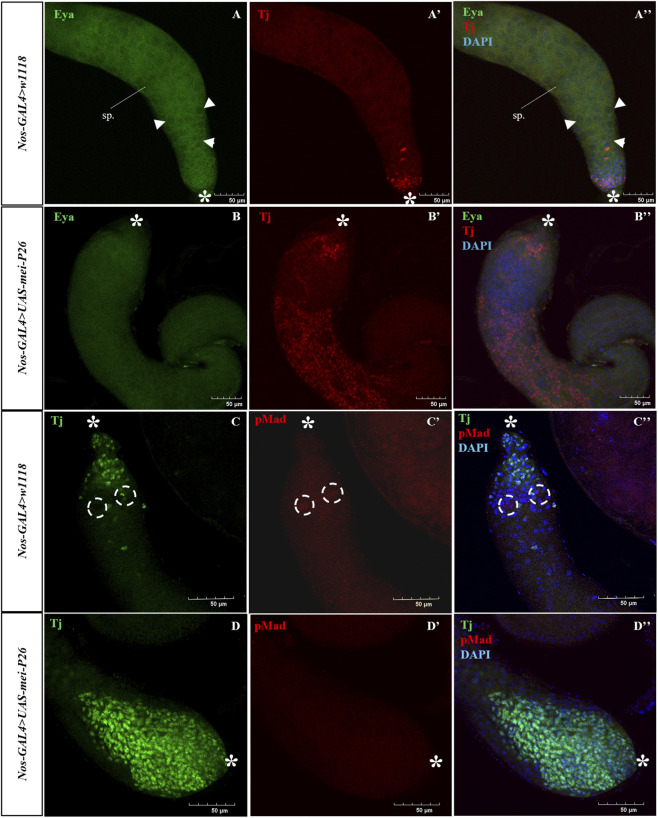
Germline *mei-P26* overexpression lacks differentiated somatic cells with the absence of BMP signaling. **(A–B”)** Early transit amplifying (TA) somatic cells are marked by traffic jam (Tj) (in red) and late differentiated somatic cells are marked by Eyes of absent (Eya) (in green). **(B–B”)** *Nos>UAS-mei-P26* has proliferation of Tj+ somatic cells and lacks differentiated Eya-marked somatic cells compared to control **(A–A”)**. These testes lack BMP signaling marked by pMad (in red) **(D–D”)**, although there is evident proliferation of Traffic jam (Tj) marked (in green) somatic cells compared to control **(C–C”)**. Arrowhead showing Eya staining around the spermatocytes, sp., and dotted circles marking the pMad marked region. Image frame: 50μm. * indicates the apical tip of the testis.

To confirm the defective differentiation of somatic cells, the truncated testes were stained with Tj to mark somatic cells at TA division stage of testis and Eyes absent (Eya) for differentiated somatic cells supporting meiotic spermatocytes. As expected, germline-specific *mei-P26* overexpression resulted in the absence of Eya-positive cells, indicating a failure to generate spermatocytes and to enter meiotic division (Figures 3B–B"), in contrast to control testes, which exhibited a well-defined transition of somatic cells from Tj-positive to Eya-positive states (Figure 3A–A”), consistent with normal differentiation of spermatogonia into spermatocytes (sp). Mei-P26 expression is high in germline stem cells (GSCs) and decreases as bam expression increases until meiosis. During differentiation, bam is suppressed while *mei-P26* levels increase in spermatocytes (Insco et al., 2012; Samuels et al., 2024).

Previous studies have shown that loss of BMP signaling in germline stem cells leads to upregulation of *bam*, and that *bam* and *mei-P26* function in a closely linked regulatory pathway controlling exit from transit-amplifying (TA) divisions (Kawase et al., 2004; Salerno-Kochan et al., 2022). Based on these findings, we examined whether BMP signaling is altered in response to germline-specific *mei-P26* overexpression by analyzing the distribution of phosphorylated Mad (pMad) in the apical region of the testis. In control testes, strong pMad staining was detected in germline stem cells surrounding the hub, consistent with active BMP signaling required for stem cell maintenance (Figure 3C’). In contrast, testes overexpressing *mei-P26* in the germline exhibited a near-complete loss of detectable pMad signal in the apical region (Figure 3D’). This reduction in BMP signaling was accompanied by disorganization of somatic cells and the absence of fusome structures, indicating disruption of the germline niche and impaired maintenance of early germ cells. Together, these findings suggest that germline-specific *mei-P26* overexpression suppresses BMP signaling, potentially through ectopic elevation of *bam* in early germ cells, thereby contributing to germline stem cell depletion and testis truncation.

### Mei-P26 overexpression in the germline triggers apoptosis

**FIGURE 4:**
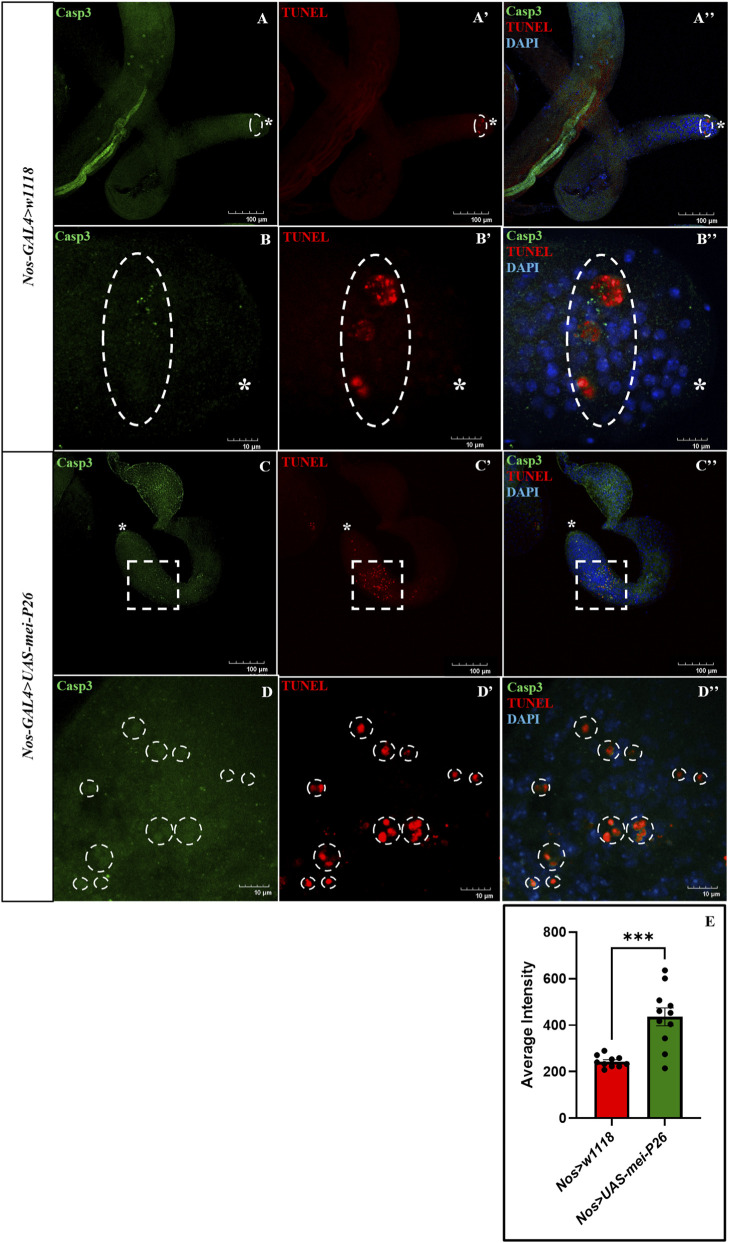
Overexpression of germline-specific *mei-P26* induced apoptosis in the testes. Active cleaved Caspase3 marking early apoptosis (green) and TUNEL staining marking late apoptosis induced DNA fragmentation (red). **(A–A”)** Apoptosis in the apical tip of the testis with a few spermatogonia going through apoptosis. **(B–B”)** Magnification of the apoptotic TUNEL-positive region of the apical tip. **(C–C”)** *Nos>UAS-mei-P26* testis has elevated TUNEL-positive apoptotic cells at the mid-stage of testis. **(D–D”)** Magnification of the TUNEL-positive region of the truncated testis. **(E)** Quantification of TUNEL intensity in control, normal, and germline *mei-P26* overexpressed truncated testis. Image frame: **(A–A”, C–C”)** 100 μm, (B–B”, D–D”) 10μm. ‚ indicates the apical tip of the testis.

Although truncated testes displayed extensive proliferation of Tj-positive somatic cyst cells (Figure 2B), these cells did not exhibit phospho-Histone H3 (PH3) staining (Figure 1B), indicating a lack of mitotic activity and suggesting a possible block in differentiation. To determine whether this absence of PH3 signal was associated with cell death, we performed TUNEL assays to detect apoptotic cells. Strikingly, truncated testes exhibited a high level of TUNEL-positive signal (Figures 4C,D) compared to control (Figures 4A,B), indicating widespread apoptosis as quantified by increased TUNEL fluorescence intensity (Figure 4E). These observations suggest that, in the absence of germline cells, somatic cyst cells fail to receive survival cues and undergo apoptosis, ultimately contributing to the formation of the truncated testis phenotype. Consistent with this, previous studies have shown that germline-derived signals, particularly those mediated through the EGFR and JAK/STAT pathways, are essential for cyst cell survival and differentiation, and disruption of these germline-to-soma interactions leads to apoptosis and testis degeneration (Amoyel, et al., 2016b; Kiger et al., 2001; Sarkar et al., 2007; Schulz et al., 2002).

Notably, apoptotic cells exhibited distinct spatial patterns between conditions. In control testes, apoptosis occurred predominantly within clustered spermatogonia (Figure 4B’), as previously reported (Lu and Yamashita, 2017). In contrast, germline-specific *mei-P26* overexpressing testes displayed apoptotic events in isolated individual cells lacking organized clusters (Figure 4D’). This failure to form clustered spermatogonia is consistent with the absence of branching fusomes observed in *mei-P26* overexpressing testes (Figure 2D), indicating an early defect in germ cell mitotic entry and cyst formation that likely underlies the dispersed pattern of apoptosis.

Together, these findings suggest that while somatic cyst cells and germline cyst cells initially proliferate, they ultimately undergo apoptosis due to the loss of germline-derived survival signals from spermatogonial cysts, culminating in the truncated and degenerative phenotype of the testis.

### Somatic Mei-P26 overexpression drives an opposite cell fate

Mei-P26 overexpression in gonadal somatic cells results in a phenotype distinct from that observed with germline-specific *mei-P26* overexpression, as it leads to impaired testis development in males. Although somatic cell specific *mei-P26* overexpression induces lethality in males, the recovered unmated female progenies had enlarged ovaries (Supplementary Figure S3) (Steenwinkel et al., 2022), opposite to what has been observed with the germline-specific *mei-P26* overexpression with rudimentary ovaries (Supplementary Figure S1). To circumvent this lethality in males, *tubulin-Gal80* ^*ts*^ was used to suppress the expression of Gal4 until the eclosion of adult male flies in 18 °C and these flies were collected and transferred to 29 °C to express Gal4 to drive the expression of *mei-P26* in testicular somatic cells. To evaluate whether this lethality is due to *mei-P26* overexpression alone, *mei-P26* was knocked down in the background of this somatic cell specific overexpression using *mei-P26*-RNAi line. The male-specific lethality was rescued, confirming that somatic cell-specific *mei-P26* overexpression alone is sufficient to induce this male-specific lethality (Supplementary Figure S2C).

**FIGURE 5:**
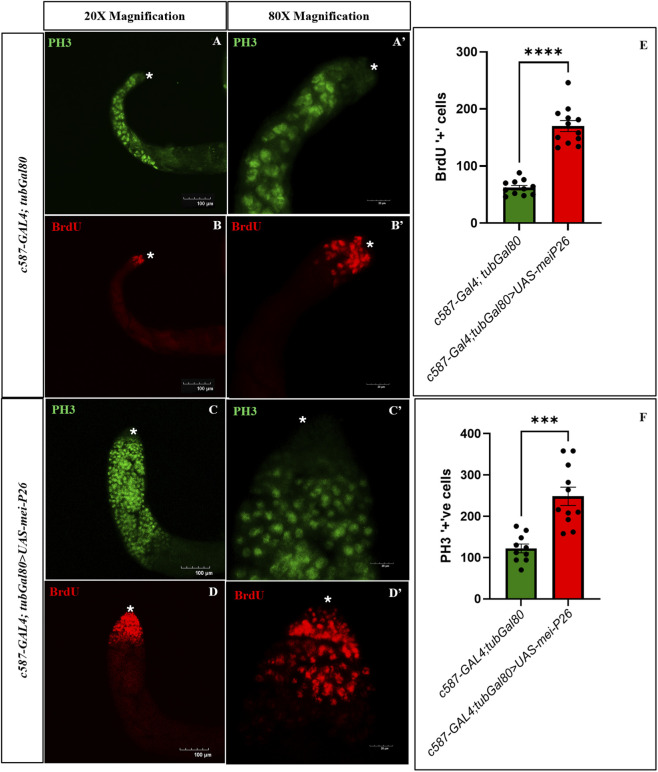
Unlike in the germline, somatic *mei-P26* overexpression adopts an alternate fate. Cells at mitotic and meiotic cell division stained with Phospho-histone H3 (PH3, green) and cells at S-phase of cell-cycle during TA division are marked by BrdU (red). **(A–B’)** Control testis with BrdU and PH3 showing cell proliferation and differentiation respectively. **(C–D’)** Somatic-cell specific *mei-P26* overexpression has elevated BrdU positive proliferating cells along with PH3 positive differentiating cells. **(E)** Number of BrdU positive proliferating cells are significantly higher in the somatic cell *mei-P26* overexpressed testes compared to control. **(F)** Number of PH3 positive mitotic and meiotic differentiating cells are significantly higher in the mutant testes compared to control. (A’–D’) X80 magnification of the **(A–D)** images. Image frame: **(A–D)** 100 μm, **(A’–D’)** 20 μm. * indicates apical tip of the testis.

Surprisingly, overexpression of *mei-P26* in somatic cells resulted in a strikingly different phenotype compared to germline overexpression. In *c587>UAS-mei-P26* testes, a marked increase in the number of cells in S-phase was observed, as indicated by increased incorporation of BrdU throughout the apical region (Figure 5D) compared to the control testis (Figure 5B) with BrdU only incorporated in the apical tip of the testis (Figure 5B’). In addition, the number of cells that had entered mitosis was dramatically elevated, with abundant phospho-histone H3 (PH3)-positive cells (Figure 5C) compared to controls (Figure 5A).

This increase in both BrdU and PH3 positive cells reflects a robust elevation in proliferation, with more cells actively cycling through S and M phases (Figures 5E,F). Moreover, these proliferating cells also showed signs of rapid progression beyond S-phase, rather than cell-cycle arrest and germline depletion. Together, these findings reveal that *mei-P26* overexpression in somatic cells drives a hyperproliferative, differentiation committed cells, representing a sharp contrasting fate to the proliferative arrest and germline loss observed when *mei-P26* is overexpressed in germ cells.

### Somatic *mei-P26* overexpression drives hyperproliferation and TOR activation

**FIGURE 6:**
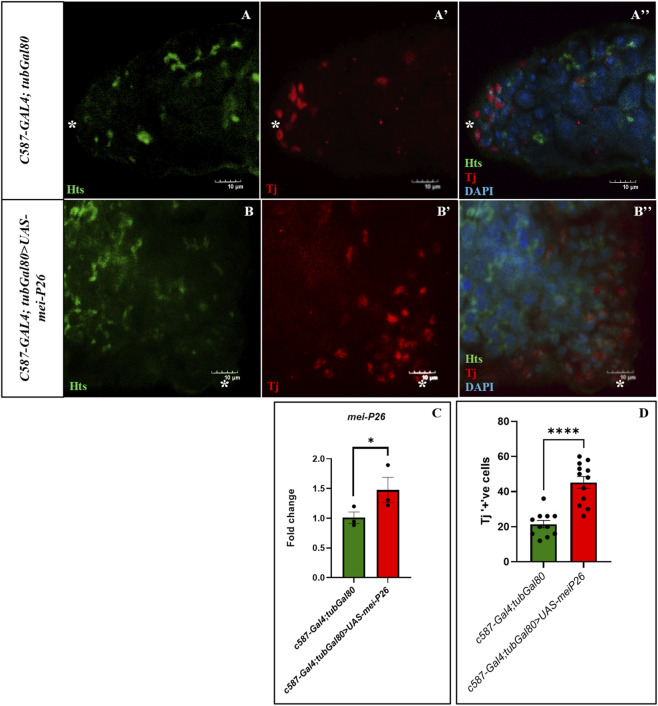
Somatic *mei-P26* overexpression induces both germline and somatic cell proliferation and differentiation. Fusomes interconnecting germline cells stained with Hu-li tai shao (Hts, green) and somatic cells at TA division is marked by Tj (red). **(A–A”)** Control testis with hts and Tj showing both germline and somatic cells. **(B–B”)** Somatic-cell specific *mei-P26* overexpression has increased Tj positive somatic cells along with elevated Hts positive branched fusomes indicating differentiation of germline cells. **(C)** *mei-P26* mRNA quantification of somatic cell mediated *mei-P26* overexpression under *tub-Gal80*. **(D)** Number of Tj positive somatic cells during TA division are significantly higher in the mutant testes compared to control. Image frame: **(A–B”)** 10μm. * indicates apical tip of the testis.

To determine which cell populations were actively proliferating under somatic cell-specific overexpression of *mei-P26*, markers for germline and somatic lineages were tested. Notably, both germline and somatic cells exhibited strong proliferative activity accompanied by differentiation. Germline cells displayed increased numbers of branched fusomes (Figure 6B), a hallmark of differentiating spermatogonial cysts, indicating accelerated progression from stem cells toward differentiated states as observed in controls (Figure 6A). In parallel, there was a marked increase in Tj positive somatic cells, consistent with hyperproliferation of somatic lineages (Figures 6B,D). Together, these observations suggest that, in contrast to germline-specific *mei-P26* overexpression which primarily results in germline depletion, somatic cell-specific overexpression drives hyperproliferation and differentiation of both germline and somatic cell populations. To test the efficiency of the Gal80 system, *mei-P26* expression was quantified and an elevation of *mei-P26* mRNA expression was observed in *c587-Gal4; tubGal80>UAS-mei-P26* testes compared to control (Figure 6C).

**FIGURE 7:**
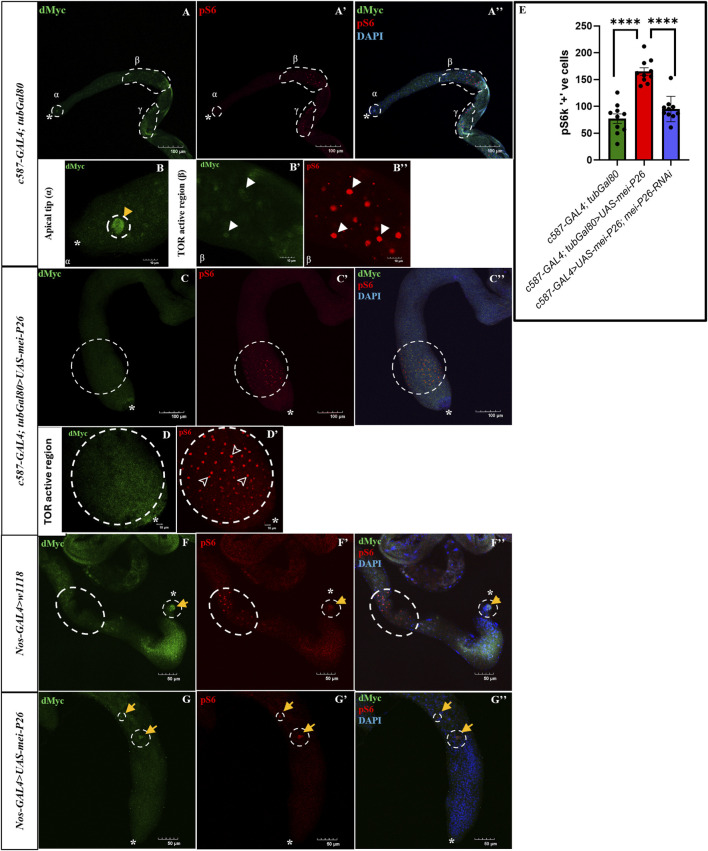
TOR signaling is elevated in somatic cell-specific *mei-P26* overexpression but suppressed in germline-specific *mei-P26* overexpression. dMyc (green) and phosphorylated S6 (pS6, red) marking TOR signaling in the **(A–A”,F-F”)** control testis **(C–C”)** somatic-cell specific *mei-P26* overexpression where pS6 positive puncta is pushed closer to the apical tip, and **(G–G”)** germline-specific *mei-P26* overexpression lacking pS6 puncta. **(A)** dMyc is present at the apical tip (α) and pS6 is enriched at the mid (β) to late (γ) region of control testis. Magnification of these regions are represented in B-B” with white arrowheads showing pS6 positive puncta. **(C)** Somatic *mei-P26* overexpression with elevated pS6 positive puncta marked with dotted circle and a magnification of this region **(D–D’)** which lacks dMyc but enriched with pS6 (arrowheads). **(F–G”)** Dotted circles indicating region with pS6 and yellow arrowheads indicating dMyc staining. **(E)** Number of pS6 positive cells significantly higher in the mutant testes compared to control indicting activated TOR signaling in the somatic cell mediated *mei-P26* overexpression and this phenotype is rescued with *mei-P26* knockdown. Image frame: **(A–A”, C–C”)** 100 μm, **(B–B”, D–D’)** 10 μm, **(F–G”)** 50 μm. * indicates apical tip of the testis.

Next, to determine whether the hyperproliferation and differentiation observed with somatic cell-specific *mei-P26* overexpression are linked to TOR pathway activation, the distribution of pS6, a marker of mTORC1 activity involved in the TOR signaling pathway, was examined. A marked increase in pS6 signal intensity compared to controls was observed, indicating elevated TOR activity (Figures 7D’,E). Interestingly, this pS6 signal elevation was rescued with *mei-P26* knockdown, suggesting suppression of Mei-P26 is sufficient to both rescue the lethality of the overexpression construct and repress the elevation of pS6 signal in the testis induced by *mei-P26* overexpression (Figure 7E; Supplementary Figure S2C). Notably, the pS6 puncta appeared enriched closer to the apical tip of the *mei-P26* overexpressed testis (Figure 7C’) compared to control (Figures 7A’,B’), indicating that TOR signaling is activated early upon *mei-P26* overexpression. This elevated signaling coincided with accelerated cell-cycle entry, increased mitotic activity, and the appearance of smaller individual mitotic cells as can be seen in Figure 5C compared to control (Figure 5A), consistent with a hyper-proliferative state. These findings align with prior reports that TRIM-NHL proteins, including Mei-P26, can activate TOR signaling to promote differentiation and translational output (Gui et al., 2023).

On the other hand, pS6 puncta were almost absent (a maximum of 2-3 puncta) in the germline-specific *mei-P26* overexpression (Figure 7G’) compared to the control testis with pS6 positive puncta (Figure 7F’), indicating repressed TOR signaling in the germline cells, similar to what has been observed previously in germline-specific TOR knockdown (Basar et al., 2019; Clémot et al., 2024).

Interestingly, even though the TOR signaling pathway activity was elevated in somatic cell-specific *mei-P26* overexpressed testes, the dMyc signal was absent (Figures 7C,D). In contrast, the control testis showed a clear dMyc signal in the apical tip region (Figures 7A,G). This finding diverges from the usual positive correlation between TOR and Myc in proliferative tissues. The phenotype supports previous reports indicating that *mei-P26* antagonizes dMyc by repressing its expression, thereby preventing excessive growth. (Herranz et al., 2010). The smaller cell size observed in somatic *mei-P26* overexpression during TA endoreplication also supports the previously noted phenotype in dMyc mutants, which resulted in reduced cell size during endoreplication in ovarian follicle cells. (Tamori and Deng, 2013). Thus, Mei-P26 appears to promote TOR-driven proliferation that favours differentiation while suppressing Myc activity.

Additionally, ectopic overexpression of *mei-P26* caused lethality in both males and females, while ectopic knockdown of *mei-P26* did not induce lethality. Although germline-specific *mei-P26* knockdown did not result in lethality, spermatocyte specific *mei-P26* knockdown led to a reduction in spermatocytes (Supplementary Figure S4B) compared to controls (Supplementary Figure S4A), indicating a requirement for Mei-P26 during spermatocyte development. Despite this reduction, overall testis morphology remained largely intact. The relatively mild phenotype observed upon germline *mei-P26* knockdown (Supplementary Figure S4D) may reflect functional redundancy with other RNA-binding proteins, including Held out wings (HOW), Benign gonial cell neoplasm (Bgcn), and Tumorous testis (Tut), which are known to regulate spermatogonial differentiation, especially expression of *bam* during transit-amplifying divisions (Chen et al., 2014; Harris et al., 2025). In contrast, somatic cell-specific *mei-P26* knockdown did not produce detectable defects in testis morphology or germline organization under the conditions examined (Supplementary Figure S4F). Collectively, these findings support a model in which the phenotypes induced by *mei-P26* overexpression reflect signaling-dependent gain-of-function effects, rather than loss of *mei-P26* activity, which can be buffered by redundant RNA-binding proteins.

## Discussion

The regulation of tissue growth and homeostasis requires a finely tuned balance between proliferation, differentiation, and signaling inputs. Germline differentiation in the testis is governed by tightly regulated crosstalk between germline cells and their surrounding somatic cyst cells. Ablation or loss of somatic cells disrupts this communication and leads to the accumulation of undifferentiated germ cells, highlighting the essential role of somatic cell encapsulation in germline differentiation (Lim and Fuller, 2012). Germline-derived Spitz (Spi) activates Epidermal growth factor receptor (EGFR) signaling in the soma, which is required for proper cyst cell encapsulation and for supporting germline transit-amplifying divisions; disruption of somatic EGFR leads to defective gonialblast enclosure and accumulation of undifferentiated germ cells (Kiger et al., 2000; Tran et al., 2000). Mechanistically, somatic EGFR signaling promotes germline differentiation in part by indirectly suppressing BMP signaling, while Transforming growth factor β (TGF-β) signaling in cyst cells further restricts spermatogonial proliferation in a cell non-autonomous manner (Matunis et al., 1997; Zheng et al., 2021). In parallel, physical coupling via junctional components such as discs large (dlg) and gap junction-mediated exchange of small molecules establishes a permissive microenvironment essential for coordinated survival and differentiation of both germline and somatic cells (Papagiannouli and Mechler, 2009; Tazuke et al., 2002). However, how post-transcriptional regulators, particularly RNA-binding proteins, integrate with these signaling pathways to fine-tune germline-somatic communication remains largely unexplored.

TRIM32, the mammalian ortholog of Mei-P26, regulates stem cell fate and proliferation, and its dysregulation has been linked to both tumor suppression and tumor promotion depending on cellular context (Izumi and Kaneko, 2014; Liu et al., 2014). It is often elevated in various types of human cancers and this elevation is correlated to radiation resistance and poor prognosis, resulting in treatment relapses in patients, making it a suitable candidate for targeted therapies (Cui et al., 2016; Wang et al., 2018; Xu et al., 2022). In this study, a cell type-specific role of an RNA-binding protein, Mei-P26 was uncovered in regulating cellular homeostasis of *Drosophila* testis. By independently overexpressing *mei-P26* in germline or somatic cells, a distinct cell-dependent outcomes were revealed which highlights how cell lineage determines the functional output of this tumor-suppressor protein. When *mei-P26* was overexpressed in the germline, germline loss due to loss of BMP signaling from the niche, S-phase cell-cycle arrest, absence of mitotic entry, and increased apoptosis were observed, resulting in truncated testes. This suggests that elevated Mei-P26 disrupts the differentiation potential of germline and somatic cells, possibly by interfering with cell cycle progression and survival. These results are consistent with earlier reports describing the role of Mei-P26 in regulating the balance between stem cell maintenance and differentiation (Neumüller et al., 2008; Li et al., 2012). Within this context, Mei-P26 may act as a restrictive regulator that prevents low-quality germ cells from continuing through spermatogenesis.

In striking contrast, somatic cell-specific overexpression of Mei-P26 produced the opposite phenotype. Robust proliferation of both germline and somatic cells, along with elevated mitotic activity. These findings suggest that the somatic lineage acts as a proliferative driver when Mei-P26 is upregulated, stimulating both self-expansion and non-cell-autonomous germline proliferation. The contrasting outcomes highlight that Mei-P26 function is not fixed but context-dependent, shaped by lineage-specific cues and regulatory networks, consistent with its broad role as a post-transcriptional regulator (Ferreira et al., 2014; Joly et al., 2013; N. Liu et al., 2009; Rastegari et al., 2020; Salerno-Kochan et al., 2022).

Precocious Mei-P26 expresion in GSC/GBs (via nos-GAL4) collapses the germline lineage and yields truncated testes that resemble ectopic bam phenotypes. In normal testes, BMP (Gbb/Dpp) signals from the niche maintain GSCs in part by repressing bam transcription; when BMP is reduced, bam rises and GSCs are lost (Kawase et al., 2004). Consistent with this axis, Bam is sufficient to drive premature differentiation and deplete the stem-cell pool when misexpressed in early germ cells (Ohlstein and McKearin, 1997; Samuels et al., 2025). Here, therefore we propose that early Mei-P26 tilts the Bam cassette toward differentiation, establishing a positive feedback loop that prematurely exhausts GSCs and phenocopies nos > bam. However, this elevation of bam, arrests the cells at endoreplication stage during late TA division where bam along with bgcn suppress *mei-P26* expression until terminal differentiation (Insco et al., 2012; Li et al., 2003). In this scenario, the suppression of *mei-P26* remains persistent due to the elevated Bam, mirroring the previously observed *mei-P26* mutant phenotype in the testis (Insco et al., 2012). As this elevation cannot be suppressed, the cells are unable to exit mitosis and enter terminal differentiation (Samuels et al., 2025).

Mechanistically, this proliferative surge in somatic cells was accompanied by a marked increase in pS6 signal, indicating increased activity of the TOR signaling pathway. Notably, pS6 puncta were enriched at the closer to the apical tip. This spatial localization implies that Mei-P26 promotes TOR pathway activity specifically within the transit amplifying endoreplication region to promote early differentiation. This observation aligns with recent work demonstrating that Mei-P26, along with other TRIM-NHL proteins, can directly activate Tor kinase to enhance translational output during differentiation (Gui et al., 2023). Our data extend this mechanism to the testis, demonstrating that Mei-P26 modulates TOR signaling in a spatially and lineage-restricted manner.

Interestingly, despite robust TOR activation, dMyc was not upregulated in the somatic Mei-P26 overexpression background. Since TOR and Myc typically act synergistically to promote growth, their uncoupling implies selective modulation by Mei-P26. Prior studies have shown that Mei-P26 represses Myc expression to prevent excessive proliferation (Ferreira et al., 2014; Herranz et al., 2010). Our results support this model, Mei-P26 activates TOR signaling to promote differentiation-coupled proliferation while simultaneously repressing Myc to prevent uncontrolled growth. The uncoupling of TOR activation from Myc induction observed upon somatic *mei-P26* overexpression parallels regulatory states described in cancer cells, in which mTOR signaling can drive growth independently of MYC to avoid oncogenic stress and apoptosis (Fumarola et al., 2005). Thus, Mei-P26 enforces a controlled proliferative program that preserves tissue integrity, particularly critical in the testis, where coordination between somatic and germline cells is essential for spermatogenesis and niche homeostasis (Lim et al., 2012; Tamirisa et al., 2018).

**FIGURE 8:**
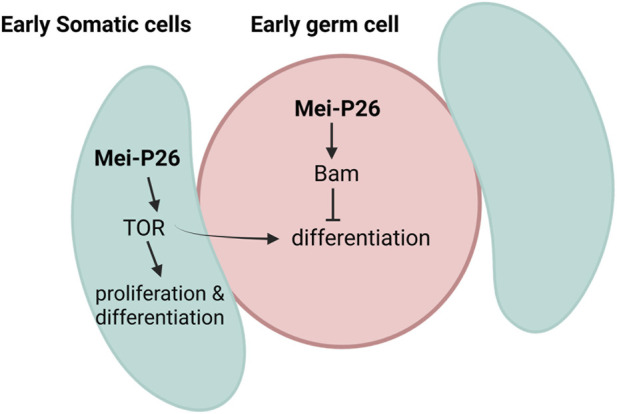
Model of how Mei-P26 modulates dual role in cell-specific manner. Overexpression of *mei-P26* in the early germline cells, induces misexpression of *bam* which exhausts the germline stem cell pool and arrest cells at transit amplifying endoreplication stage. Without the repression of *bam* by HOW, *bam* accumulates and prevents entry into mitotic differentiation. On the other hand, *mei-P26* overexpression in the somatic cells induces elevation of TOR which causes cell proliferation and promotes premature mitotic differentiation. Elevation of TOR in the somatic cell also commits the mitotic differentiation of the germ cells.

The mechanistic basis of somatic-germline coordination mediated by Mei-P26, we note that prior work in the *Drosophila* ovary has demonstrated a functional link between Mei-P26, TOR signaling, and *bam* dependent differentiation (Gui et al., 2023), underscoring *Drosophila* as a tractable model for tumor-relevant stem cell dysregulation. Our findings reveal that Mei-P26 exerts distinct, cell type-specific control over proliferation and differentiation within the testis. Germline overexpression predominantly causes loss of germ cells, S-phase arrest, rapid apoptosis, and truncated testes, indicative of a proliferative blockade and compromised tissue maintenance, likely arising from ectopic bam induction in early germ cells. In contrast, somatic overexpression promotes coordinated hyperproliferation of both somatic and germline populations, accompanied by elevated differentiation activity. This proliferative coupled differentiation shift is correlated with enhanced and apically enriched pS6 signaling, reflecting elevation of the TOR activity (Figure 8).

Collectively, these results identify Mei-P26 as a dual-functional growth regulator that can toggle between restricting and amplifying differentiation in a cell type-dependent manner, potentially through differential modulation of signaling pathways within the same tissue. Together, our findings establish the *Drosophila* testis as a genetically tractable model to dissect how context-dependent post-transcriptional regulation by TRIM-NHL proteins contributes to tissue homeostasis and disease-relevant growth control mechanisms. These regulatory principles are broadly relevant to stem cell dysregulation in cancer and other disorders of aberrant growth and differentiation. Future studies will be directed toward elucidating the mechanistic relationship by which somatic TOR signaling influences germline bam expression and cellular behavior.

## Data Availability

The original contributions presented in the study are included in the article/Supplementary Material, further inquiries can be directed to the corresponding author.

## Supplementary material

SUPPLEMENTARY FIGURE S1 Germline *mei-P26* overexpression in female ovaries induced rudimentary ovaries. Mitotic PH3 (green) and S-phase cell cycle (BrdU, red) marking cell proliferation in **(A–A”)** control ovaries **(B–B”)** germline-cell specific *mei-P26* overexpression where elevated BrdU signal is observed with less PH3 positive cells in the rudimentary ovaries. Arrowhead and dotted circles showing BrdU positive region in both control and mutant respectively. Image frame: **(A–B”)** 250 μm.

SUPPLEMENTARY FIGURE S2 Germline and somatic *mei-P26* overexpression-induced phenotypes were rescued with *mei-P26* knockdown. **(A–A’)** pS6 (red) indicating TOR signaling in the rescued testis. **(B)** Quantification of the percentage of normal testis in control, nos>UAS-mei-P26, and nos>UAS-mei-P26; mei-P26-RNAi testes. **(C–C’)** Somatic *mei-P26* overexpression rescue with *mei-P26* RNAi suppresses elevated pS6 puncta observed in the *mei-P26* overexpression alone. A dotted circle showing the pS6 marked region of the testis. Image frame: **(A–A’, C–C’)** 100 μm. * indicates the apical tip of the testis.

SUPPLEMENTARY FIGURE S3 Somatic *mei-P26* overexpression in female ovaries induces ovarian enlargement. Mitotic PH3 (green) and S-phase cell cycle (BrdU, red) marking cell proliferation in **(A–A”)** control ovaries **(B–B”)** somatic-cell specific mei-P26 overexpressed ovaries. **(C)** The size of unmated female ovaries and **(D)** the length of mature eggs in the ovaries were quantified 5 days after eclosion. Arrowhead showing BrdU-positive region of the control ovaries. Image frame: **(A–B”)** 250 μm.

SUPPLEMENTARY FIGURE S4 Loss of *mei-P26* does not markedly disrupt testis morphology or induce defects in spermatogenesis. Caspase-3 (Casp3) marking apoptosis, specifically waste bags, along with α-Spectrin (α-Spec) marking fusomes in green, and Mei-P26 marking spermatocytes in red. **(A–A”)** Control testis showing Mei-P26 (in red) in spermatocytes, and **(B–B”)** spermatocyte-specific *mei-P26* knockdown showing reduced Mei-P26-positive spermatocytes in the testis. **(C–C”)** Control testis showing Mei-P26 in spermatocytes (in red) and branched fusomes (in green), meanwhile, in germline-specific *mei-P26* knockdown, no aberrant testicular phenotype was observed. **(E–E”)** Control testis with Mei-P26 spermatocytes (in red) and **(F–F”)** somatic cell-specific *mei-P26* knockdown showing almost absent Mei-P26-positive spermatocytes, although there was no aberrant halt in spermatogenesis. Image frame: **(A–F”)** 200 μm. * indicates the apical tip of the testis.
