# Supplementary figures and images for: Divergent functions of the RNA-binding protein, Mei-P26 in germline and somatic lineages of Drosophila testis
Source: Front Cell Dev Biol. 2026 Feb 18;14:1739650. doi: 10.3389/fcell.2026.1739650 (PMC12956707; doi:10.3389/fcell.2026.1739650)

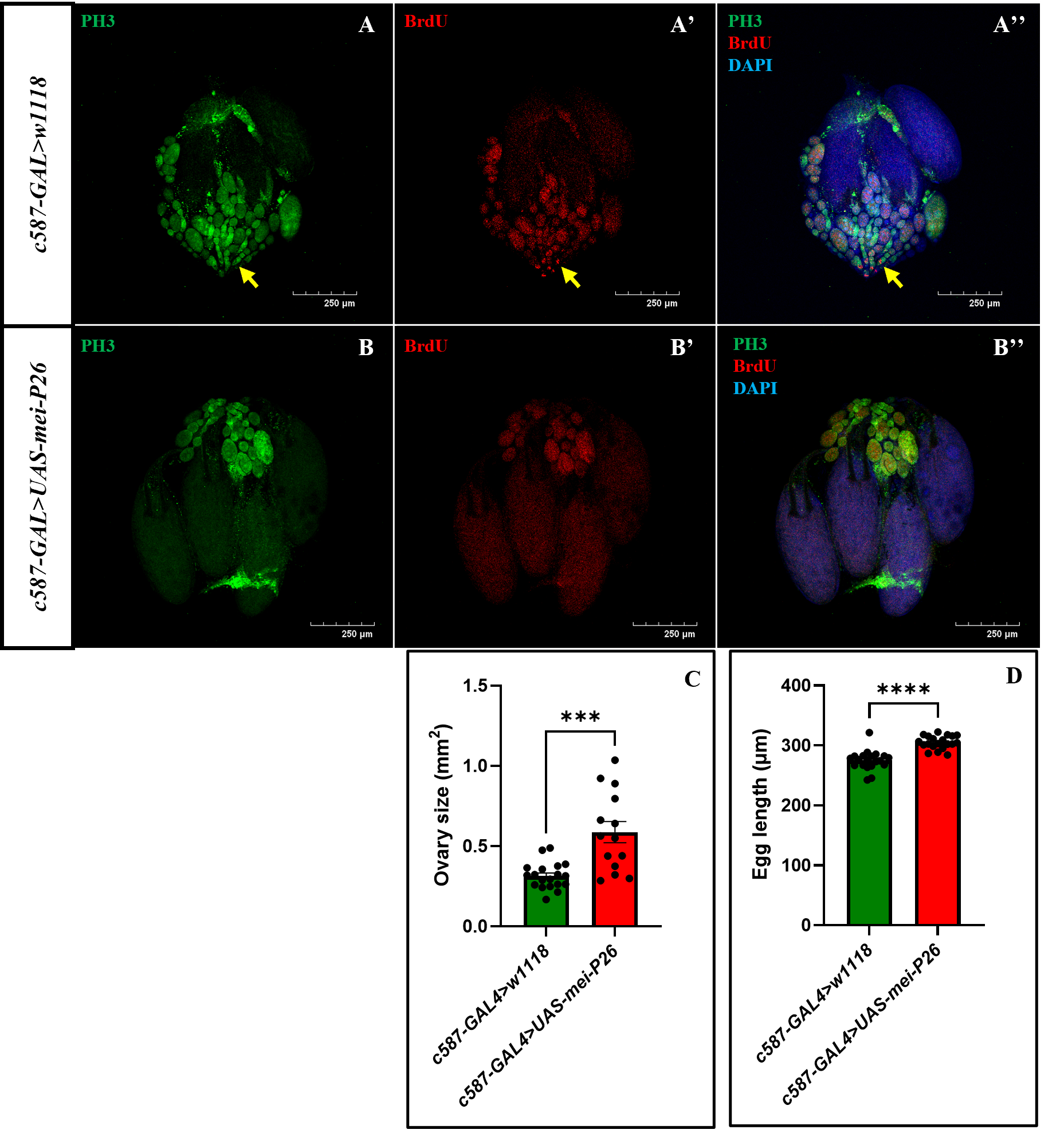

Supplement: Supplementary file 1 [file Image3.tif]

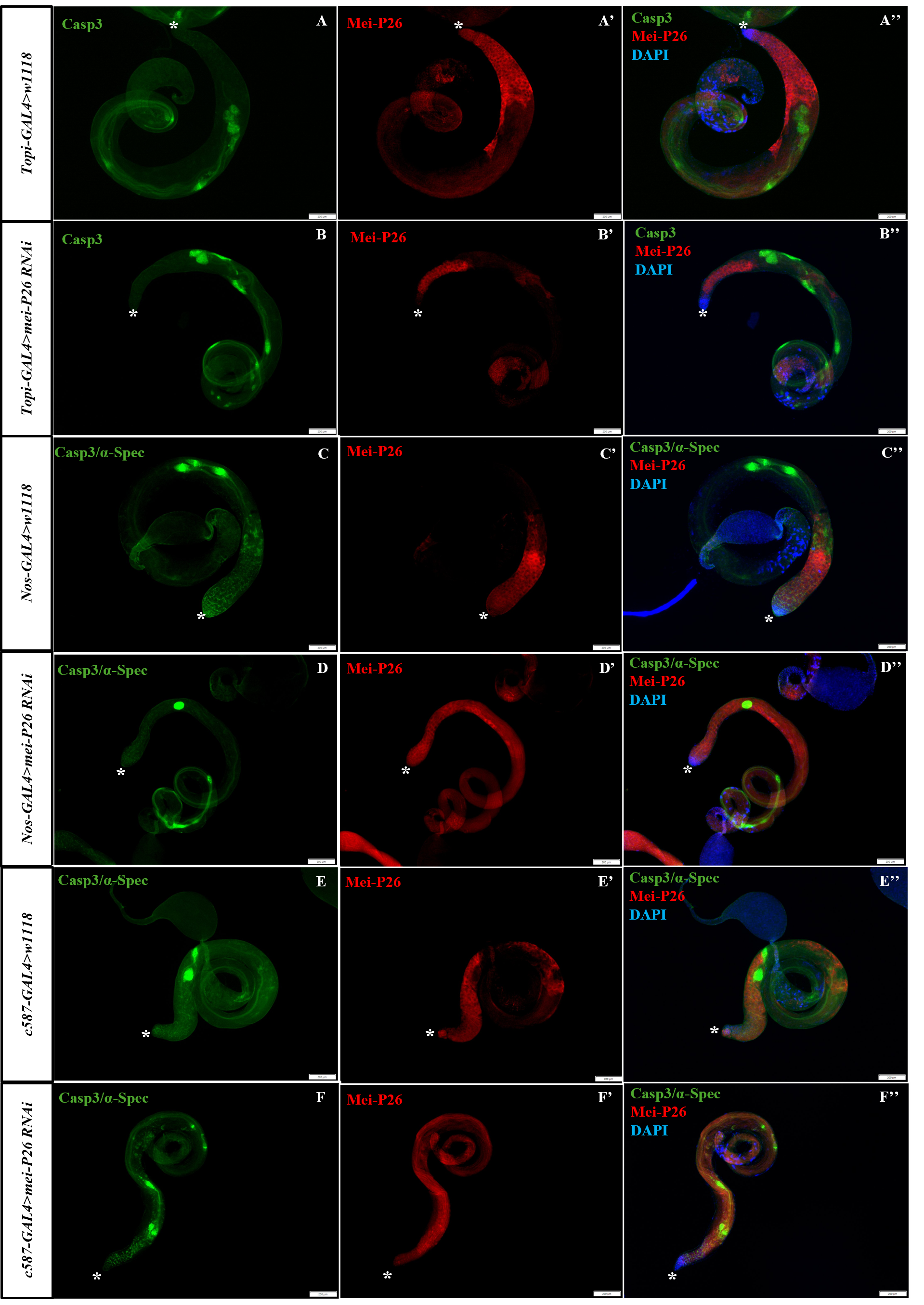

Supplement: Supplementary file 2 [file Image4.tif]

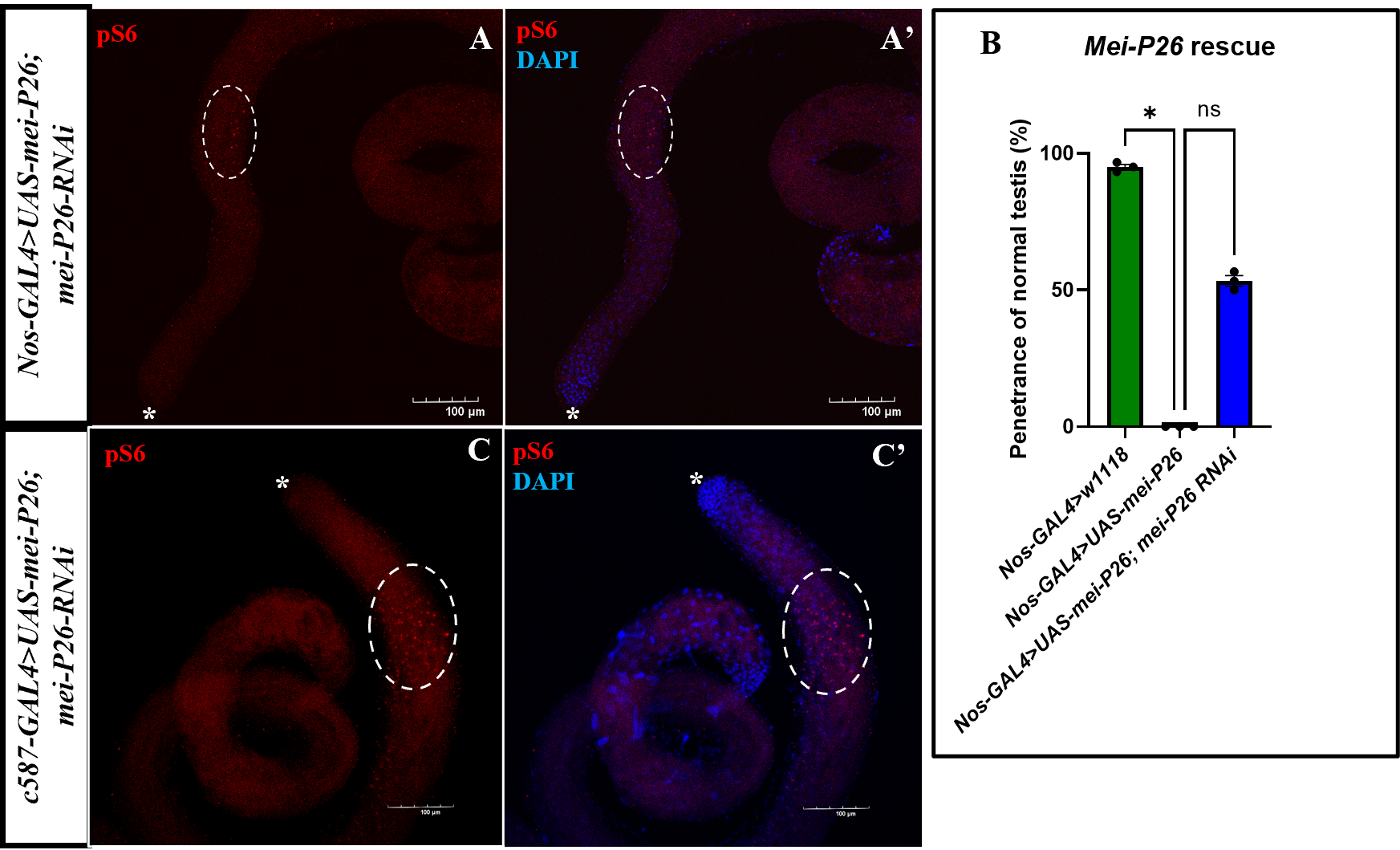

Supplement: Supplementary file 3 [file Image2.tif]

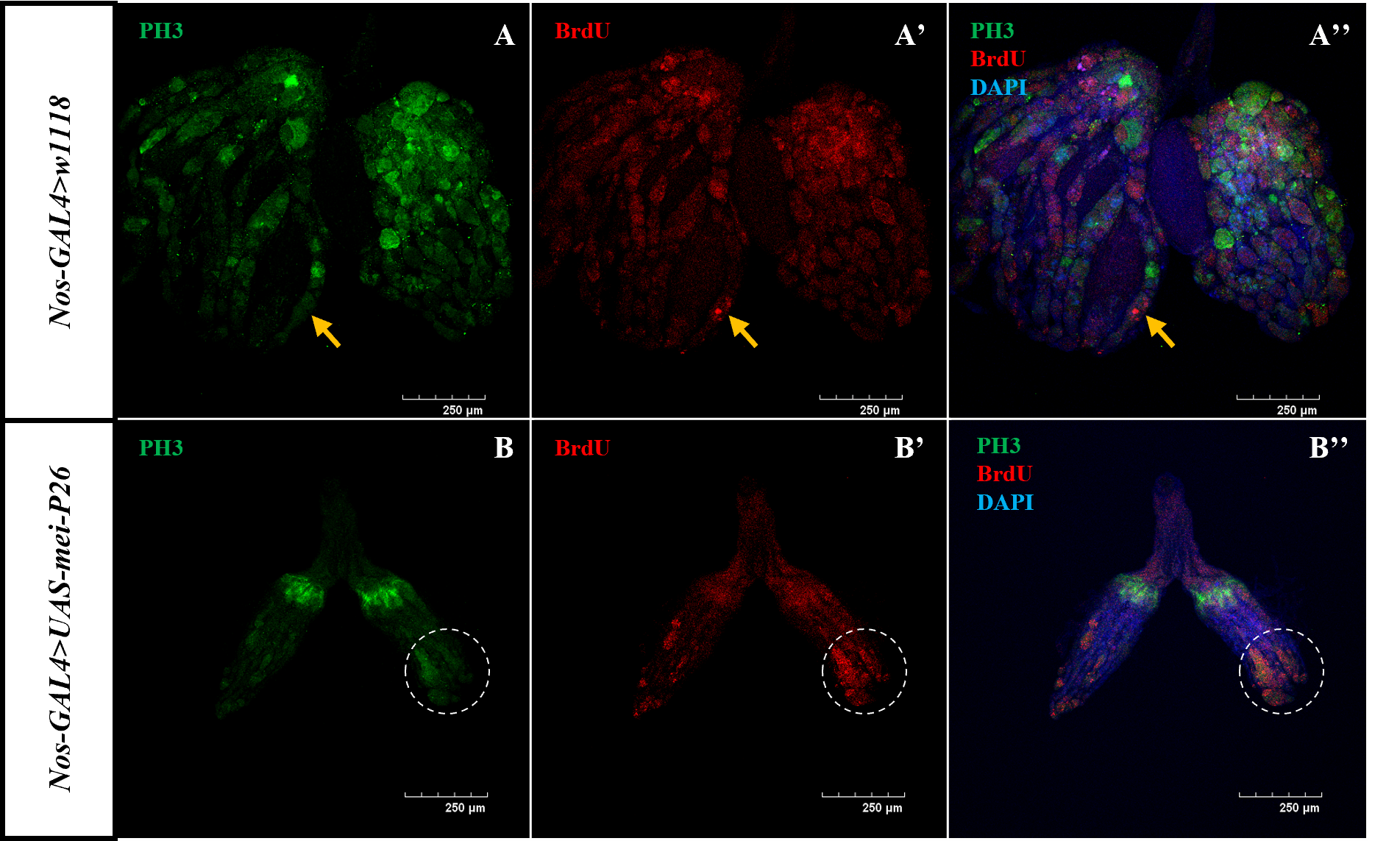

Supplement: Supplementary file 4 [file Image1.tif]
